# *Deionococcus proteotlycius* Genomic Library Exploration Enhances Oxidative Stress Resistance and Poly-3-hydroxybutyrate Production in Recombinant *Escherichia coli*

**DOI:** 10.3390/microorganisms11092135

**Published:** 2023-08-23

**Authors:** Seul-Ki Yang, Soyoung Jeong, Inwoo Baek, Jong-il Choi, Sangyong Lim, Jong-Hyun Jung

**Affiliations:** 1 Radiation Biotechnology Division, Korea Atomic Energy Research Institute, Jeongeup 56212, Republic of Koreasaylim@kaeri.re.kr (S.L.); 2 Graduate School of Biotechnology and Institute of Life Science and Resources, Kyung Hee University, Yongin 17104, Republic of Korea; 3 Department of Food and Animal Biotechnology, Seoul National University, Seoul 08826, Republic of Korea; 4 Department of Biotechnology and Bioengineering, Chonnam National University, Gwangju 61186, Republic of Korea; choiji01@chonnam.ac.kr; 5 Department of Radiation Science and Technology, University of Science and Technology, Daejeon 34113, Republic of Korea

**Keywords:** *Deinococcus proteolyticus*, oxidative stress, response regulator, PHB

## Abstract

Cell growth is inhibited by abiotic stresses during industrial processes, which is a limitation of microbial cell factories. Microbes with robust phenotypes are critical for its maximizing the yield of the target products in industrial biotechnology. Currently, there are several reports on the enhanced production of industrial metabolite through the introduction of *Deinococcal* genes into host cells, which confers cellular robustness. *Deinococcus* is known for its unique genetic function thriving in extreme environments such as radiation, UV, and oxidants. In this study, we established that *Deinococcus proteolyticus* showed greater resistance to oxidation and UV-C than commonly used *D. radiodurans*. By screening the genomic library of *D. proteolyticus*, we isolated a gene (*deipr_0871*) encoding a response regulator, which not only enhanced oxidative stress, but also promoted the growth of the recombinant *E. coli* strain. The transcription analysis indicated that the heterologous expression of *deipr_0871* upregulated oxidative-stress-related genes such as *ahp*C and *sod*A, and acetyl-CoA-accumulation-associated genes via *sox*S regulon. Deipr_0871 was applied to improve the production of the valuable metabolite, poly-3-hydroxybutyrate (PHB), in the synthetic *E. coli* strain, which lead to the remarkably higher PHB than the control strain. Therefore, the stress tolerance gene from *D. proteolyticus* should be used in the modification of *E. coli* for the production of PHB and other biomaterials

## 1. Introduction

*Escherichia coli* is one of the most common industrial hosts used as microbial cell factories for producing pharmaceuticals [1], biopharmaceuticals [2], fine chemicals [3], recombinant proteins, and biofuels [4,5]. To maximize the yield of bacterial metabolites, *E. coli* is subjected to high-cell-density fermentation [6] under aerobic conditions [7]. However, the high oxygen concentration and simultaneous accumulation of products such as fuels and chemicals can lead to diverse stresses, especially oxidative stress, which resulted in reduced industrial production [8,9]. Oxidative stress is induced by reactive oxygen species (ROS), such as hydroxyl radical (OH^·^), hydrogen peroxide (H_2_O_2_), and superoxide anion (O_2_^−^), through biological respiration including metabolic pathway destruction and DNA alteration [10]. Therefore, oxidative stress resistance is a critical factor in commercial strains, and various metabolic engineering techniques have been applied to enhance their robustness against oxidative stress [11].

Engineering regulatory factors, cell membranes, and biosynthetic pathways were employed to improve stress tolerance in *E. coli* [12], for example, the heterologous expression of the putative response regulator DRH632 from Antarctic bacteria into *E. coli* [13], the random mutation of cAMP receptor protein [11], and the overexpression of iron exporters, FetA and FetB, into *E. coli* [14]. As *Deinococcus* sp. is known for its ability to survive against multiple stresses such as γ- radiation, UV-C, oxidative stress, and DNA damage reagents, several studies have investigated its genes [15]. Heterologous expression of *Deinococcus* genes in *E. coli* successfully leads to strains with multiple stress resistance. For example, the cold-shock-domain-containing protein PprM [16], pyrroloquinoline-quinone (PQQ) synthase [17], manganese (Mn) transporter protein (MntH), and a small heat shock protein (Hsp20) confer oxidative stress tolerance to *E. coli* [18,19]. Moreover, *dr_1558*, a response regulator, confers on *E. coli* multiple stress resistances to oxidative, acidic, salt, and heat stresses [20]. Interestingly, some reports suggest that introducing *Deinococcal* genes into other hosts promotes not only oxidative stress resistance but also its metabolic activity. For instance, the *Deinococcal* gene *irrE* (*pprI*) improves the growth and ethanol production of *Zymomonas mobilis* [21], the proliferation of *E. coli* [18], and lactic acid production in *Lactococcus lactis* [19]. In addition, *dr_1558* enhances the production of succinic acid [22], γ-aminobutyric acid (GABA) in *E. coli* [23], and cadaverine in *Corynebacterium glutamicum* [24]. Moreover, the biosynthesis of poly(3-hydroxybutyrate) (PHB), a biopolymer that can replace petroleum-based plastics [25], was highly produced in synthetic *E. coli* strains under *dr_1558* regulation.

To date, 67 *Deinococcus-*type strains have been isolated and sequenced. However, most studies have focused on the *D. radiodurans* genes. Recently, Lim et al. reported that complicated stress resistance systems were widely located in the genome of *Deinococcus* as conserved and divergent forms [26], which are not fully understood yet.

In this study, to confer oxidative stress resistance and cellular robustness on *E. coli*, we explored the genomic library of *Deinococcus proteolyticus.* Through a series of screening steps, we selected a gene that has the potential to resist a high concentration of hydrogen peroxide. The selected gene was introduced subsequently to the PHB-generative *E. coli* strain to investigate its impact on PHB synthesis and metabolism.

## 2. Materials and Methods

### 2.1. Bacterial Strains and Plasmids and Culture Conditions

*Deinococcus proteolyticus* MRP (taxid:693977) was used as the genomic DNA donor strain and was obtained from the Korean Agricultural Culture Collection (KACC) of the Rural Development Administration. The bacterial strains *Escherichia coli* XL1-Blue (*E. coli* recA1 endA1 gyrA96 thi-1 hsdR17 supE44 relA1 lac F’ proAB lacI^q^ZΔM15 Tn10 [Tet^r^]) was employed for the construction of a library, evaluation of survival rate under oxidative stress conditions, and fermentation for producing PHB. Plasmid pKMCAB (pKM212-MCS + pCnCAB) for PHB biosynthesis constructed previously [25] was introduced into XL1-Blue.

### 2.2. Screening of Oxidative-Resistant Clones Using the Genomic DNA Library

To construct the gDNA library of *D. proteolyticus* MRP, total DNA (1136 ng/µL) was extracted and smeared into 3–5 kb fragments. The fragments were then ligated into a modified pC31 vector provided by Macrogen (Seoul, Republic of Korea). The libraries for screening were cultured for 18 h at 37 °C with Bacto Luria-Bertani (LB) broth containing 50 µg/mL kanamycin and different concentrations of hydrogen peroxide (H_2_O_2_). The oxidative tolerance survival study was performed using exponential-phase cultures. Subsequently, another survival study to confirm *deipr_0871* function isolated from the screening was executed as described previously using the *E. coli* strains harboring pRad-0871 and pRadGro. The surviving fraction was calculated by dividing the number of colonies in the samples by the number of colonies in the controls.

### 2.3. Construction of Deipr_0871 Expression Vector

The plasmid pRadGro was used as a vector for gene cloning and protein expression of the oxidative-stress-resistance gene, *deipr_0871*. The gene was amplified from the selected clones using primer Deipr_0871_F: 5-GAGGGCCCATGAACCGTTCCCAAGCTTCTCTTG-3′ and Deipr_0871_R: 5′-GATTAAGCTTCTAGTCCAGCAGACCGATGGTG-3′. A polymerase chain reaction was performed using n*Pfu-Forte* DNA polymerase (Enzynomics, Daejeon, Republic of Korea) under the following conditions: initial denaturation at 95 °C for 5 min, followed by 30 cycles of denaturation at 95 °C for 30 s, annealing at 60 °C for 30 s, and extension at 72 °C for 45 s; followed by a final extension step at 72 °C for 1 min 30 s. 

The amplified fragments were digested with *Apa* I and *Hind* III, then ligated to pRadGro vector treated with the same restriction enzymes. The resulting plasmid was transformed into *E. coli*, XL1-Blue, to create the pRad-0871 strain. The *E. coli* strain harboring the empty vector pRadGro was used as a control strain in this study.

### 2.4. Determination of Growth and Survival of the Recombinant E. coli Strains

Cell growth was analyzed with the optical density (OD_600_) using a UV spectrophotometer (GENESYS 150 UV-visible spectrophotometer; Thermo Scientific, Waltham, MA, USA). All stress survival studies were performed using cells in the exponential phase after adjustment to approximately 10^7^ CFU/mL (OD_600_ ≈ 0.1). For the γ-radiation tolerance assay, *Deinococcus* strains were irradiated to different doses (3~15 kGy) of γ-radiation using a ^60^Co-gamma irradiator (Advanced Radiation Technology Institute, Jeongeup, Republic of Korea), then serially diluted in TGY media (0.5% Tryptone, 0.3% Yeast extract and 0.1% glucose), and spotted on TGY agar. To investigate the UV-C tolerance of *D. radiodurans* and *D. proteolyticus*, they were irradiated with 800 J/m^2^ UV-C using a UV-C crosslinker (CX-2000 UV Crosslinker, UVP, Milwaukee, CA, USA) after successive dilution and dropping on a TGY plate. For oxidative stress survival, the *Deinococcus* strains were serially diluted in TGY broth and spotted onto TGY containing 0.4 mM of hydrogen peroxide (H_2_O_2_), then incubated at 30 °C for 36 h. The recombinant *E. coli* strains introducing pRadgro and pRad-0871 were also tested with the same process at 37 °C for 18 h as described above using LB and a 0.2 mM H_2_O_2_ LB plate. The surviving fraction was calculated by dividing the number of colonies in each sample by the number of cells in the control group. 

### 2.5. Real-Time PCR (qPCR)

All sampling for RNA extraction was performed in the exponential phase of the *E. coli* strains with and without deipr_0871 under non-stressed and stressed conditions (0.45 mM H_2_O_2_), and without and with a high concentration of glucose (30 g/L). Total RNA was extracted using the RiboEx reagent (GeneAll, Seoul, Republic of Korea) supplied with DNase, and purified using the RNeasy Mini Kit (Qiagen, Hilden, Germany) according to the manufacturer’s instructions. RNA quality and concentration were estimated by measuring the optical densities of the solutions at 260 and 280 nm using a GeneQuant Pro instrument (Amersham Pharmacia Biotech, Amersham, UK). RNA (1 µg) from each sample was used for cDNA synthesis with random hexamers using a PrimeScript first-strand cDNA synthesis kit (TaKaRa Bio Inc., Kusatsu, Japan). RT-qPCR amplification was performed using SYBR Premix *Ex Taq* (TaKaRa) and an Eco Real-Time PCR system (Illumina Inc., San Diego, CA, USA) according to the manufacturer’s instructions. The housekeeping gene *polA* was used as an internal control. Primers used for RT-qPCR are listed in Table S1. All reactions were repeated in duplicates, and the relative expression was calculated using the relative quantification method.

### 2.6. Genome Analysis

The genome sequences of *D. radiodurans* R1 (GCF_020546685.1) and *D. proteolyticus* MRP (GCF_020546685.1) were obtained from the NCBI database, respectively. The two genome assemblies were examined to confirm if those qualities exceeded the proposed cutoff (over 95% completeness and under 5% contamination) using the CheckM software v1.1.6 [27]. The amino acid sequences and corresponding annotated gff files were obtained by executing the Prokka (v. 1.14.6) annotation pipeline, with the default parameters [28]. Then, the coded in-house pipeline was executed for obtaining the percentage of core and accessory COG category ratio, where DIAMOND sequence alignment (50% percent identity) and COG database were implemented [29,30]. From the annotated protein information, a donut plot depicting the positions of core and accessory genes of those two genomes was generated by using in-house code.

To predict the function of genes, the conserved domains (CDD) such as Pfam and the COG group were investigated using the InterPro database (https://www.ebi.ac.uk/interpro/, accessed on 3 April 2023).

### 2.7. Cultivation for PHB Production

To cultivate *E. coli* strains carrying the vector pKMCAB containing the PHB generative operon, seed cultures were cultivated at 30 °C in 5 mL LB medium for 18 h with shaking at 200 rpm. For the main shake flask cultures, 3% (*v*/*v*) seed cultures were added to 30 mL of LB medium (in 250 mL shake flask) supplemented with 30 g/L glucose. The cells were grown at 30 °C for 60 h at 200 rpm. Kanamycin (50 µg/mL) and ampicillin (100 µg/mL) were added to the culture medium to maintain plasmid stability. To measure glucose and PHB concentrations, growth sampling was performed at intervals of 12 h before 24 h cultures and at intervals of 6 h after 24 h.

### 2.8. Measurement of Amount of PHB

To determine the PHB concentration and glucose consumption, the cells were harvested via centrifugation at 10,000× *g* for 15 min. Cell pellets were dried in an 80 °C dry oven for 24 h, and the dry cell weight (DCW) was determined. The PHB concentration was analyzed using gas chromatography (SHIMADZU GC-2010 Plus, Shimadzu Inc., Kyoto, Japan) equipped with a flame ionization detector (FID) after solvent extraction using 3% (*v*/*v*) H_2_SO_4_ in methanol [31]. To determine the concentration of glucose consumption, cell supernatants were analyzed using high-performance liquid chromatography (HPLC; Agilent Technologies Inc., Santa Clara, CA, USA) equipped with a refractive index detector (RID).

### 2.9. Sequence Alignment

The amino acid sequences of the NarL/FixJ family (Deipr_0871, DR_1558: AAF11120, DRH577: OF298141, DRH632: LY802646, DRH1601, *E. coli* NarL: CAA48935, *E. coli* UhpA: YP002389147, *E. coli* NarP: CAD6002661, *E. coli* UvrY:CAD6011216, *E. coli* RcsB:CAD6002904, *E. coli* EvgA:CAD6007853, *Bordetella pertussis* BvgA:NP880570, *Chromohalobacter salexigens* EupR:ABE58223, *Sinorhizobium meliloti* FixJ:CAA79898) used in this study were obtained from NCBI (http://ncbi.nim.nih.gov/, accessed on 8 May 2023). Multiple sequence alignment was performed using Clustal Omega [32]. The conserved regions were visualized using GeneDoc 2.7 (Free Software Foundation, Inc., Boston, MA, USA). A phylogenetic tree was constructed using MEGA 11 based on the neighbor-joining method and evaluated using a bootstrap test with 1000 replicates [33].

## 3. Results

### 3.1. The Oxidative Stress Resistance Properties of Deinococcus Proteolyticus

**Figure 1:**
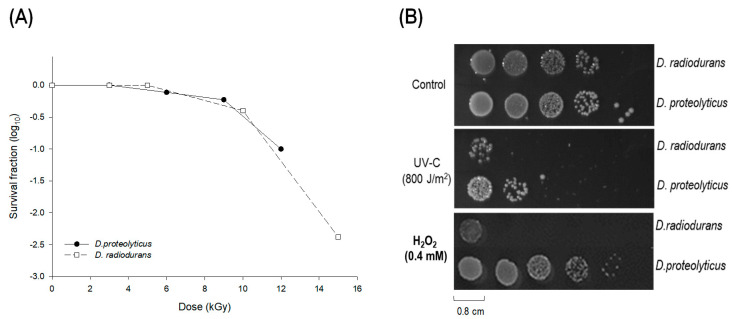
Stress resistance properties of *Deinococcus proteolyticus* and *D. radiodurans*. (**A**) Radiation resistance of *D. proteolyticus* (filled circle) and *D. radiodurans* (empty square). The cells were exposed to different doses of ^60^Co γ-radiation, then the cultures were serially diluted and were spotted onto TGY plates. (**B**) Survival fraction of *D. proteolyticus* and *D. radiodurans* against UV-C (800 J/m^2^) and oxidant (0.4 mM H_2_O_2_). The cells were serially diluted and spotted on the TGY plate, after that the plate was exposed under UV-C stress. For oxidative stress resistance test, the diluted cells were spotted on the TGY plate containing H_2_O_2_ 0.4 mM. The survival fraction was calculated by comparing to the controls under non-stress conditions.

*D. proteolyticus*, isolated from the feces of Lama glama in 1973, produces orange pigments and exhibits γ-radiation resistance similar to that of *D. radiodurans* [34]. To date, only the genome of *D. proteolyticus* has been sequenced and its stress resistance properties have not been studied [34]. To investigate *D. proteolyticus*’s resistance potential under various environmental conditions, the oxidative stress, UV-C stress, and γ-radiation tolerance of *D. proteolyticus* were examined and compared to those of *D. radiodruans*. It showed that *D. proteolyticus* exhibited as strong an endurance to radiation stress as *D. radiodurans* (Figure 1A). We also found that *D. proteolyticus* displays distinct resistance to UV-C and hydrogen peroxide. As shown in Figure 1, *D. proteolyticus* displayed approximately a 10-fold increase in UV-C resistance (800 J/m^2^) compared to *D. radiodurans*. In the presence of 0.4 mM hydrogen peroxide, *D. proteolyticus* showed minimal cell loss, whereas *D. radiodurans* exhibited a 4-log cycle decrease in viability (Figure 1B).

**Figure 2:**
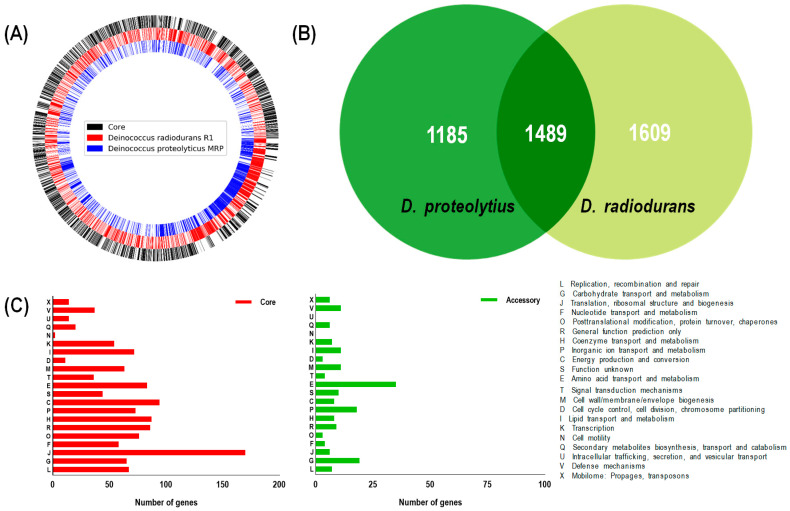
Genome comparison analysis between *D. proteolyticus* and *D. radiodurans*. (**A**) Pie chart showing the distribution of the core and accessory genes present in the pan-genome of *D. proteolyticus* and *D. radiodurans.* (**B**) Venn diagram showing the number of core and accessory genes in *D. proteolyticus* and *D. radiodurans*, respectively. (**C**) Functional analysis of core genes and accessory genes in *D. proteolyticus* using COG database.

To explain the distinct stress resistance properties of *D. proteolyticus*, we compared the genomes of *D. proteolyticus* and *D. radiodurans*. The genome of *D. proteolyticus* was reported in 2012 and consists of one chromosome (CP002536.1) and four plasmids with a GC content of 65.6%. It contains a total of 2790 genes, of which 2688 are protein-coding. ANI analysis showed that *D. proteolyticus* had a 73.25% similarity to *D. radiodurans*. A comparative analysis showed that 1489 genes were homologous (core genome) between *D. proteolyticus* and *D. radiodurans*, while 1185 were *D. protelyticus*-specific (>50%) (Figure 2). Most of the core genome was involved in translation and energy-production-associated COG groups (Figure 2C), while high diversity was observed in the amino acid metabolism and inorganic ion metabolism COG groups. Most of the known stress-resistance-associated genes, including *ddrO* and *pprI* in *D. radiodurans,* were conserved in *D. proteolyticus* [26].

### 3.2. Screening Genomic Libraries of D. proteolyticus for Genes Improving Oxidative Stress Resistance to E. coli

To identify the oxidative-stress-associated genes in *D. proteolyticus*, we constructed a genomic library using 3~5 kb gene fragments. The isolation step was implemented under varying hydrogen peroxide concentrations (1.0–1.5 mM in 1st step and 1.5–2.0 mM in 2nd step). In the first step, 446 clones showed faster growth than the wild-type (WT) strain. In the second step, 50 clones exhibited stress resistance to hydrogen peroxides (Supplementary Figure S1). The candidate clones were treated with 20 mM hydrogen peroxide for 1 h, of which two exhibited much higher tolerance phenotypes (Supplementary Figure S2). The sequencing analysis showed that one clone contained the catalase gene (*deipr_2034*), while the other clone contained two genes, namely, histidine kinase (*deipr_0870*) and a response regulator (*deipr_0871*).

**Figure 3:**
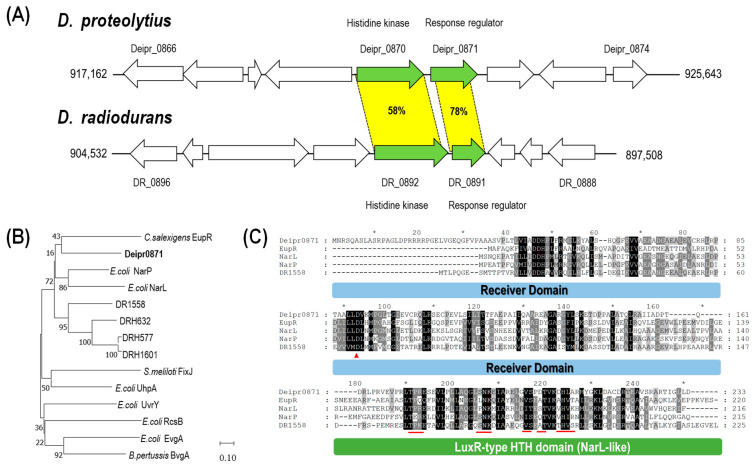
Genetic characteristics of the newly isolated response regulator, *deipr_0871*. (**A**) Genomic maps of the gene cluster of *deipr_0871* and its homolog cluster in *D. radiodurans*. Shared ORFs are connected with a green color based on sequence identity. (**B**) Phylogenetic tree of NarL/FixJ family from *D. proteolyticus* and other bacteria: *D.proteolyticus* Deipr_0871 (this study), *D. radiodurans* DR_1558 (AAF11120), *B. pumilus* DRH577 (OF298141), *Bacillus sp*. DRH632 (LY802646), *Bacillus sp*. DRH1601(LY802643), *C salexigens* EupR (ABE58223), *S. meliloti* FixJ (CAA79898), *B. pertussis* BvgA (NP880570), *E.coli* NarP (CAD6002661), *E.coli* NarL (CAA48935), *E.coli* UhpA (YP002389147), *E.coli* RcsB (CAD6002904), *E.coli* UvrY (CAD6011216), and *E.coli* EvgA (CAD6007853). (**C**) Multiple sequence alignment of Deipr_0871 and related response regulators. Domain function was predicted base on the interPro database (https://www.ebi.ac.uk/interpro/, accessed on 8 May 2023). The red arrow indicates the phosphorylation site and the DNA binding site is marked by red lines. The blue box indicated receiver domain area, whereas green box showed LuxR type HTH domain area.

Catalase encoded by *deipr_2034* shares 86% homology with catalase (DR_1998) in *D. radiodurans* which is known as the main H_2_O_2_-scavenging enzyme in *D. radiodurans*. The amino acid sequence of *deipr_0871* showed high similarity (78%) to that of *dr_0891* in *D. radiodurans*. However, its paired protein, DR_0892, showed only 58% similarity with Deipr_0870 (Figure 3A). Moreover, the neighboring genes of this cluster exhibited properties distinct from those of *D. radiodurans* (Supplementary Table S2).

To further characterize *deipr_0871*, we analyzed its domain composition using the InterPro database (https://www.ebi.ac.uk/interpro/, accessed on 8 May 2023). The response regulator Deipr_0871 included an N-terminal receiver domain and a LuxR C-like DNA-binding helix-turn-helix (HTH) domain (Figure 3C). CDD domain analysis indicated that Deipr_0871 is a response regulator of the NarL-like family. A phylogenetic tree with the well-known NarL/FixJ family of response regulators indicated that Deipr_0871 was independently located with *C. salexigens* EupR and closed with *E. coli* NarP and NarL response regulators (Figure 3B). Especially, the sequence of Deipr_0871 was so far from that of DR_1558 which was also classified into NarL-like family response regulator in *D. radiodurans*. The alignment analysis showed that Deipr_0871 has a relatively short linker region between the receiver domain and the HTH domain. Although the phosphorylation site (D91) was highly conserved, the putative DNA binding sites of Deipr_0871 differed slightly (Figure 3C).

### 3.3. Effect of Deipr_0871 on the Oxidative Stress Resistance of E. coli Strain

**Figure 4:**
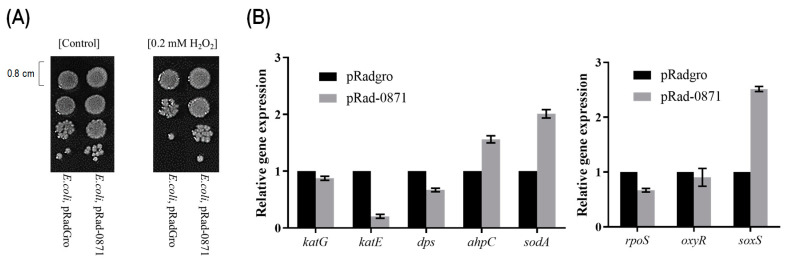
Effect of Deipr_0871 on oxidative stress resistance. (**A**) Confirmation of the oxidative stress resistance of pRad-0871 strains via drop assay on 0.2 mM H_2_O_2_ LB plates compared to the *E. coli* strain harboring an empty vector (pRadgro). (**B**) Transcriptional changes in major oxidative-stress-responsive genes: *katG, katE, dps, ahpC,* and *sodA* (**left**), and oxidative stress regulators: *rpoS, oxyR*, and *soxS* (**right**) in strains pRadgro and pRad-0871.

To investigate the effect of *deipr_0871* on the oxidative stress resistance in *E. coli,* we constructed an auto-inducible expression vector, pRad-0871, and transformed it into the XL1-blue strain. The stress resistance potential of *E. coli* in the presence of hydrogen peroxide was examined using an LB medium containing 0.2 mM hydrogen peroxide. Under exposure to hydrogen peroxide, the cell count of *E. coli* in the control group was shown to be 10-fold lower than *E. coli* with *deipr_0871* (Figure 4A).

We then assessed the expression of genes associated with oxidative stress in *E. coli*, including *katG* and *katE* (catalases), *dps* (DNA-binding protein from starved cells), *ahpC* (subunit C of alkyl hydroperoxide reductase), and *sodA* (superoxide dismutase) [35], using real-time PCR (qPCR) (Figure 4A). The expression of *sodA* was upregulated (2-fold increase) in the *E. coli* strain harboring *deipr_0871* than that of the control strain, whereas the mRNA levels of *katG*, *katE*, and *dps* were not increased in the presence of *deipr_0871*. The main stress response sigma factor *rpoS* was unaffected by *deipr_0871.* Additionally, we examined the expression of two crucial *E. coli* genes, *oxyR,* and *soxS,* that encode regulators of the oxidative stress response [36]. The transcript levels of *oxyR* were comparable in both the control and *deipr_0871* expression strains. However, *soxS* transcript levels increased 2.5-fold in the presence of *deipr_0871* compared to that of the control strain (Figure 4B).

### 3.4. Effect of Deipr_0871 on Growth of E. coli Strain

**Figure 5:**
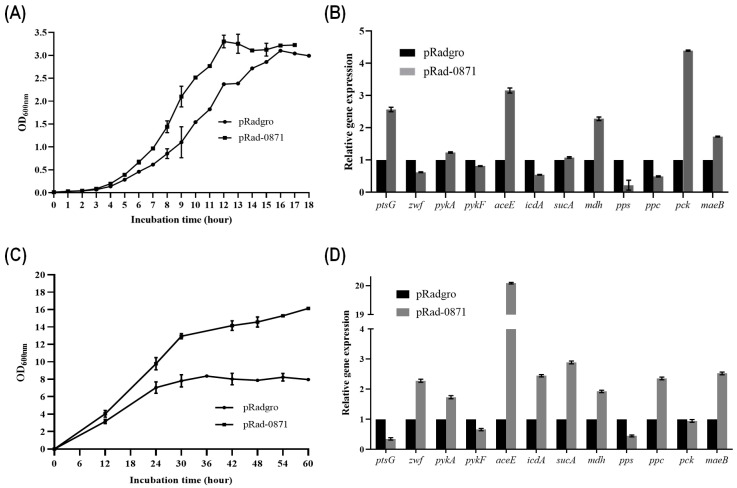
Effect of Deipr_0871 on growth of *E. coli* strain supplemented without or with high glucose (30 g/L). (**A**) Determination of growth curve of *E. coli* strain without or with *deipr_0871*. (**B**) The relative transcription levels of genes involved in central carbon metabolism in strains pRadgro and pRad-0871. (**C**) Determination of growth curve of E. coli strain without or with deipr_0871 in a high concentration of glucose supplementation (**D**) The relative transcription levels of genes involved in central carbon metabolism in a condition with a high concentration of glucose.

We found that *deipr_0871* affects not only oxidative stress resistance but also the growth rate in *E. coli*. Although the optical cell density of *E. coli* harboring *deipr_0871* was not different from that of the control, the growth of the *E. coli* strain harboring *deipr_0871* in the exponential phase was 1.4 times faster than that of the control strain (Figure 5A).

To examine changes in the metabolic ability of *E. coli* strain with *deipr_0871*, transcripts of 11 genes related to the central carbon pathway in *E. coli* were measured using qPCR (Figure 5B). Most genes were not affected by *deipr_0871; icdA, pps,* and *ppc* expression was downregulated 2-fold, 5-fold, and 2-fold, respectively. *pck* and *mdh* transcripts, encoding sequential enzymes that generate PEP from malate through oxaloacetate, increased by 4.5-fold and 2.3-fold, respectively. Moreover, *aceE* (pyruvate dehydrogenase E1) converting pyruvate to acetyl-CoA was upregulated 3-fold in the *E. coli* strain with *deipr_0871* compared to that in the control, which showed acetyl-CoA accumulation.

Interestingly, the addition of glucose to the medium significantly promoted growth (Figure 5C). The optical density at 600 nm (OD_600nm_) was 16.12 ± 0.34 in *deipr_0871*-harboring strains, which was 2-fold higher than that of the control strain (7.96 ± 0.58). The transcripts of *zwf*, *icdA*, and *sucA*, which contribute to the energy cycle in the pentose phosphate pathway and tricarboxylic acid (TCA) cycle, were upregulated 2.3-fold, 2.4-fold, and 3-fold, respectively. Especially, the transcript of *aceE* was highly enhanced by 20-fold in the presence of glucose (Figure 5D).

### 3.5. Effect of Deipr_0871 on Valuable Metabolite, PHB, Production in the E. coli Strain

To use these properties to produce the valuable metabolite PHB, we introduced PHB synthesis genes *pha*A, *pha*B, and *pha*C, into the *E. coli* strain using the pKMCAB vector. Cell growth and glucose consumption rates were significantly higher in *deipr_0871*-overexpressing strains than in controls. After 60 h of incubation with *E. coli*, up to 80% of the glucose was consumed in both strains. Table 1 reveals that the DCW of *E. coli* carrying pKMCAB and pRad-0871 reached 3.88 ± 0.09 g/L by consuming 26.06 ± 0.81 g/L glucose, whereas that of the control strain only reached 1.96 ± 0.05 g/L with a glucose consumption of 27.62 ± 1.79 g/L. In addition, PHB production was increased from 0.731 ± 0.03 g/L in control *E. coli* to 3.139 ± 0.07 g/L in *E. coli* with pKMCAB and pRad-0871 (Table 1).

## 4. Discussion

In this study, we found that *D. proteolyticus* exhibited high resistance potential toward oxidative stress. In the core-genome analysis between *D. proteolyticus* and *D. radiodurans*, important genes for stress resistance were observed in both strains with high similarity, which was also observed in the comparative analysis of 11 genomes in the *Deinococcus* species [26].

TCSs facilitate the detection of environmental signals and regulate diverse stress-resistance-associated genes [37]. Among the stress response genes in *D. proteolyticus*, we revealed that *D. proteolyticus* has 11 two-component systems, 6 orphan histidine kinases, and 6 orphan response regulators. A comparative analysis with the genome of *D. radiodurans* showed that most of the response regulators (73%) have a high degree of similarity (>50%) with response regulators from *D. radiodurans* (Supplementary Figure S3). It was proposed that highly preserved response regulators may play a pivotal role in the stress response.

From a genomic library constructed using the gDNA of *D. proteolyticus*, we isolated a gene (Deipr_0871) that enhanced the oxidative stress resistance of *E. coli* strains. The selected clone contained a response regulator (RR) domain involved in a two-component signal transduction system (TCSs) with histidine kinase (HK) [37]. Deipr_0871 was composed of two domains, receiver domain and NarL-like LuxR type-HTH domain. The NarL/FixJ family has divergent roles in bacterial systems such as nitrogen fixation and sugar phosphate transport [38].

A phylogenetic tree with other known NarL family regulators revealed that Deipr_0871 was found in close proximity to *C. salexigens* EupR, *E. coli* NarP, and NarL response regulators (Figure 3). *C. salexigens* EupR was previously recognized as playing a function in compatible solute absorption, whereas NarP and NarL can regulate nitrogen metabolism. The heterologous expression of DR_1558, a NarL-like family response regulator in *D. radiodurans,* resulted in similar phenotypic properties with those of deipr_0871, such as high oxidative stress resistance, despite their low homology (38%). A previous study revealed that the oxidative stress resistance of the *E. coli* strain harboring DR_1558 was enhanced via an increase in *rpoS* transcripts. Additionally, other DR_1558 homologs in Antarctic bacteria can upregulate *rpoS* transcripts [13]. RpoS is an alternative sigma factor that plays a central role in adaptation to many suboptimal growth conditions by controlling the expression of many genes, including those affecting phenotypic traits such as metabolic pathways and the expression of genes required to survive nutrient deprivation [20].

Multiple alignments of amino acid composition located in the putative DNA binding site suggested that Deipr_0871 may control the stress resistance of *E. coli* strains in a manner different from that of other regulators. It was also confirmed by a transcriptional change in the recombinant E. coli strain in the presence of deipr_0871. The heterologous expression of *deipr_0871* did not regulate *rpoS* or its downstream genes (Figure 4B). Under normal growth conditions, SodA and SoxS transcripts were highly expressed in the presence of *deipr_0871*, which increased the stress resistance of the *E. coli* strain. In *E. coli*, two major regulatory defense systems respond to oxidative stress: *oxy*R and *sox*RS. [36]. OxyR responds to hydrogen peroxide and induces the expression of catalases such as *kat*G, *dps*, and *ahp*C, whereas SoxRS responds to redox-active compounds and regulates superoxide dismutase (SodA). [36]. SodA plays a role in decreasing the levels of cytotoxic ROS [39], which has the properties of cellular responses to oxidative stresses for detoxification [36]. Moreover, SoxS can improve NADPH pools and promote antioxidant defense by mediating the reduction of thioredoxins or glutaredoxins [40,41].

**Figure 6:**
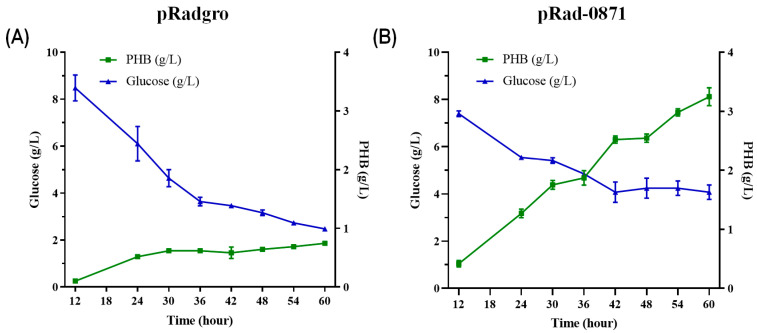
Effect of Deipr_0871 on PHB production under the glucose supplementation condition. In the aerobic condition with high concentration of glucose (30 g/L), PHB production and glucose consumption between *E. coli* strain (**A**) without and (**B**) with the *deipr_0871* gene.

According to Shery et al. and Henard et al., SoxS activates the expression of genes involved in carbon metabolic pathways, such as glycolysis and the TCA cycle [40,41]. The SoxS deletion mutant shows reduced glucose uptake and growth rates [42]. This can explain the effect of *deipr_0871* on growth, as the growth rate in the presence of *deipr_0871* was higher than that in the control strain. Interestingly, glucose supplementation altered the metabolic ability of the *E. coli* strain in the presence of *deipr_0871*, which boosts the growth rate and cell density of the *E. coli* (Figure 6). In addition, it stimulated PHB production in the recombinant *E. coli* strain. PHB is a bioplastic produced intracellularly by microorganisms to save energy as a carbon source [43,44]. The sufficient provision of acetyl-CoA and the availability of the cofactor NADPH significantly impact the synthetic efficiency of PHB, and metabolic engineering to expand the supply of NADPH has been attempted to boost PHB production [45].

**Figure 7:**
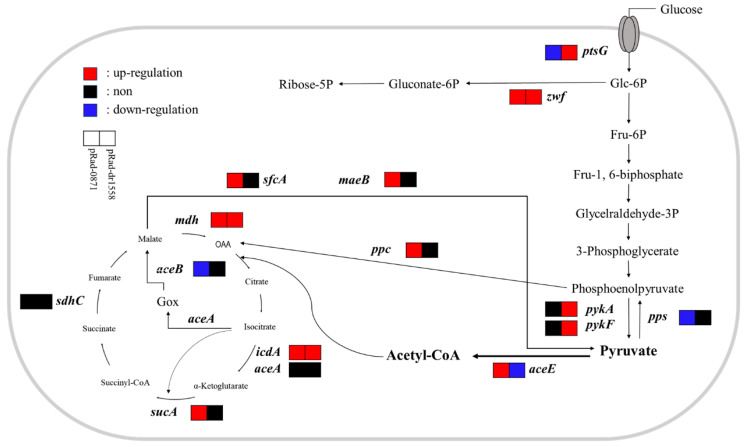
Schematic illustrating comparison of the differential expression of the energy metabolism gene between strain pRad-0871 and strain pRad-dr1558 [25].

As shown in Figure 7, this suggests that Deipr_0871 differentially regulates the central carbon metabolism compared to DR_1558. NADPH-generating genes (*zwf, icdA, sucA,* and *mdh*) in the pentose phosphate pathway and TCA cycle were upregulated in the presence of deipr_0871, indicating an improvement in cell growth by generating energy (ATP, NADPH, etc.) through the pentose phosphate and TCA pathways. The higher expression levels of genes related to the pentose phosphate pathway, glycolysis, and TCA cycle produce more energy in the form of ATP, NADH, and NADPH for cell growth, resulting in the increased proliferation period of pRad-0871 strains. High concentrations of NADPH also activate acetoacetyl-CoA reductase (*phaB*) involved in the synthesis of PHB. In addition, the overflow of glycolysis and the TCA pathway generate and accumulate numerous pyruvate and acetyl-CoA precursors of the product (PHB), which are elevated.

Using a genomic library of *D. proteolyticus*, we isolated the NarL-like response regulator Deipr_0871. The introduction of *deipr_0871* in *E. coli* showed improved oxidative stress resistance and growth rate through the soxS regulation system. This is different from the other *Deinococcal* NarL-like response regulator, DR_1558. Moreover, its mechanism in metabolic pathways and stress resistance was employed to improve PHB production by boosting the acetyl-CoA and NADPH generation pathways, respectively. It concluded that *deipr*_*0871* can also be employed to improve the capabilities of industrial strains.

## Figures and Tables

**Table 1:** Effects of deipr_0871 on cell concentration and PHB production (60 h).

Strains	DCW (g/L)	Yield (g_PHB_/g_Glucose_)	PHB Conc. (g/L)	PHB Content (wt%)
*E. coli* XL1-Blue (pKMCAB+pRad-0871)	3.88 ± 0.09	0.12 ± 0.004	3.139 ± 0.07	80 ± 3
*E. coli* XL1-Blue (pKMCAB+pRadGro)	1.96 ± 0.05	0.03 ± 0.007	0.731 ± 0.03	38 ± 1

## Data Availability

The data that support the findings of this study are available from the corresponding author upon request.

## Supplementary Materials

The following supporting information can be downloaded at: https://www.mdpi.com/article/10.3390/microorganisms11092135/s1. Table S1. List of quantitative real-time PCR primers in this study. Table S2. Genetic information of the neighbor genes of *deipr_0871* and its homolog, *dr_0891.* Figure S1. Screening of positive clones by hydrogen peroxide. *E. coli* (XL1-Blue) containing recombinant DNA fragments were incubated in liquid medium with 50 µg/mL kanamycin and spotting on 1.0, 1.5, and 2.0 mM H_2_O_2_ in 1st, 2nd screening step, 20 mM H_2_O_2_ for 1 h in 3rd screening. Figure S2. 3rd screening selection. The white arrows indicate *E.coli* harboring empty vector as control; The red arrows indicate selected E.coli harboring *D. proteolyticus* genes with outstanding oxidative stress resistance. Adjusting cell OD_600_= 0.4. Figure S3. Core and accessory analysis of two component system, orphan histidine kinase and orphan response regulators in *D. proteolyticus.* Core and accessory genes were determined based on the homology (50%) and functional domains were analyzed using interpro database.
