# Supplementary material for: Deionococcus proteotlycius Genomic Library Exploration Enhances Oxidative Stress Resistance and Poly-3-hydroxybutyrate Production in Recombinant Escherichia coli
Source: Microorganisms. 2023 Aug 23;11(9):2135. doi: 10.3390/microorganisms11092135 (PMC10538107; doi:10.3390/microorganisms11092135)
Supplement: Supplementary file 1 [file microorganisms-11-02135-s001.zip › microorganisms-2555713-supplementary.pdf]

Supplementary Material

Deionococcus proteotlycius Genomic Library Exploration Enhances Oxidative Stress Resistance and Poly-3-hydroxybutyrate Production in Recombinant *Escherichia coli*

Seul-Ki Yang [1,2], Soyoung Jeong [1,3], Inwoo Baek [1], Jong-il Choi [4], Sangyong Lim [1,5] and Jong-Hyun Jung [1,*]

[1] Radiation Biotechnology Division, Korea Atomic Energy Research Institute, Jeongeup 56212, Republic of Korea; wisdomyang@kaeri.re.kr (S.-K.Y.); soyoung@kaeri.re.kr (S.J.); inwoobaek@kaeri.re.kr (I.B.); saylim@kaeri.re.kr (S.L.)

[2] Graduate School of Biotechnology and Institute of Life Science and Resources, Kyung Hee University, Yongin 17104, Republic of Korea

[3] Department of Food and Animal Biotechnology, Seoul National University, Seoul 08826, Republic of Korea

[4] Department of Biotechnology and Bioengineering, Chonnam National University, Gwangju 61186, Republic of Korea; choiji01@chonnam.ac.kr

[5] Department of Radiation Science and Technology, University of Science and Technology, Daejeon 34113, Republic of Korea

* Correspondence: jungjh83@kaeri.re.kr; Tel: +82-63-570-3203; Fax: +82-63-570-3238

***Keywords*: **Deinococcus proteolyticus, oxidative stress, response regulator, PHB

Supple Table S1. List of quantitative real-time PCR primers in this study

**Pathway**	***Gene***	**Forward primer**	**Reverse primer**
Internal control	*polA*	GCTGAACGTGCAGCCATTAA	CAATCATCGCCCGTTTGATAA
	dnaA	TGGCGAAAGAGCTGACTAACC	ACGGCAGGCATGAAGCA
Oxidative stress related gene	*katE*	CAATTCCACAAGGGCAAAGT	CTTCCCTTCGGCATTAATCA
	katG	GCATCTGCTTCTACCTTCCG	CTCAACACCAACCACACCAG
	dps	AAACAAGCGCACTGGAACAT	CTGCACTGCACGTTCTGC
	ahpC	ACTGCAGAAACTGGGCGTAG	GTTGTCGAAGTTACGGGTCAG
	sodA	GCTGAAAGGCGATAAACTGG	ACACATCCAGGCCCATAATC
Sigma factors	*rpoS*	CGCCGCCGGATGATC	CAGACCACGATTGCCATAACG
Oxidative stress regulator	*oxyR*	GAAGCACAGACCCACCAGTT	CAAACAACGGCACTTCAATG
	soxS	TACTTGCAACGAATGTTCCG	ACATAACCCAGGTCCATTGC
Pentose phosphate pathway Glycolysis	*ptsG*	ACTGGCGTTGTGCTGTCCTT	GGTAAGCAGCCCACTGAGAGA
	zwf	GGCGCTGCGTTTTGCTAA	TTCTGCCACGGTAATCTCAACA
	pykA	GCGCTGACCGAAAAAGACAA	CAGCCAGGTAATCTACGCCAAT
	pykF	CGAGTTCAACAATGACAACCGT AA	ATCAGCGGAGCATCCAGTTT
	aceE	CCAAAGGCAAAGCGACAGTAAT	CCTGGTGCGCGATGTTTT
TCA cycle	*icdA*	AAGTTCACCGAAGGAGCGTTTA	GCCACCGTCGATCAGTTCA
	sucA	GTCTCGCGTTGCCAAGATTTAT	GTGGCGTAAGCGAGGTTTTC
	mdh	AGGCGCTTGCACTACTGTTAAAA	CGGGAGTCACTGGAGCGATA
	sdhC	CACATGATGATGGATTTTGGCTAT	GCACGACAGTAATAACAAAGGA GATTT
Gluconeogenesis and anaplerosis	*pps*	CCTCCCTGGGTGAAATGATTACTA	GGCGGTTGTGGCGAAA
	ppc	GGAAGAGAACCTCGGCTACAAA	CCGCCCATCCACGAAGT
	pck	CGTCTTTCCGTCCGTTTCAT	GCTCGGGCGAATAAACATGT
	maeB	GAAGAGCTGGCGGAGATCAC	GCGGCTCAATACCAAAACGA
	sfcA	TGCGGTTGGCAAAATGG	AGGGCTTCGGCAGAGGTT
Glyoxylate shunt	*aceA*	TGCACGGTGAGTCGAAAAAA	TAGACTGCTTCAATACCCGCTTT
	aceB	TGGCGTGGTGAGGCAAT	GGAAGAAATAGAGCGCAAAATCA

Supple Table S2. Genetic information of the neighbor genes of *deipr_0871* and its homolog, *dr_0891*

**The list of genes in *D. proteolyticus***		
**locus_tag**	**Location**	**Product**
Deipr_0866	918064..917162	hypothetical protein
Deipr_0867	919101..918133	NUDIX hydrolase
Deipr_0868	919285..919527	cold-shock DNA-binding domain protein
Deipr_0869	921102..919723	Methylenetetrahydrofolate--tRNA-(uracil-5-)-methyltransferase
Deipr_0870	921241..922314	integral membrane sensor signal transduction histidine kinase
Deipr_0871	922311..923012	two component transcriptional regulator, LuxR family
Deipr_0872	923052..923813	protein of unknown function DUF124
Deipr_0873	924924..923878	thioredoxin reductase
Deipr_0874	925053..925643	Holliday junction ATP-dependent DNA helicase ruvA

**The list of genes in *D. radiodurans***		
**locus_tag**	**Location**	**Product**
DR_0888	897508..898269	hypothetical protein
DR_0889	898277..898627	hypothetical protein
DR_0890	898628..899065	conserved hypothetical protein
DR_0891	899727..899143	DNA-binding response regulator
DR_0892	900833..899724	sensor histidine kinase
DR_0893	901689..900994	conserved hypothetical protein
DR_0894	903279..901774	hypothetical protein
DR_0895	903383..903769	hypothetical protein
DR_0896	903759..904532	ribosomal large subunit pseudouridine synthase B

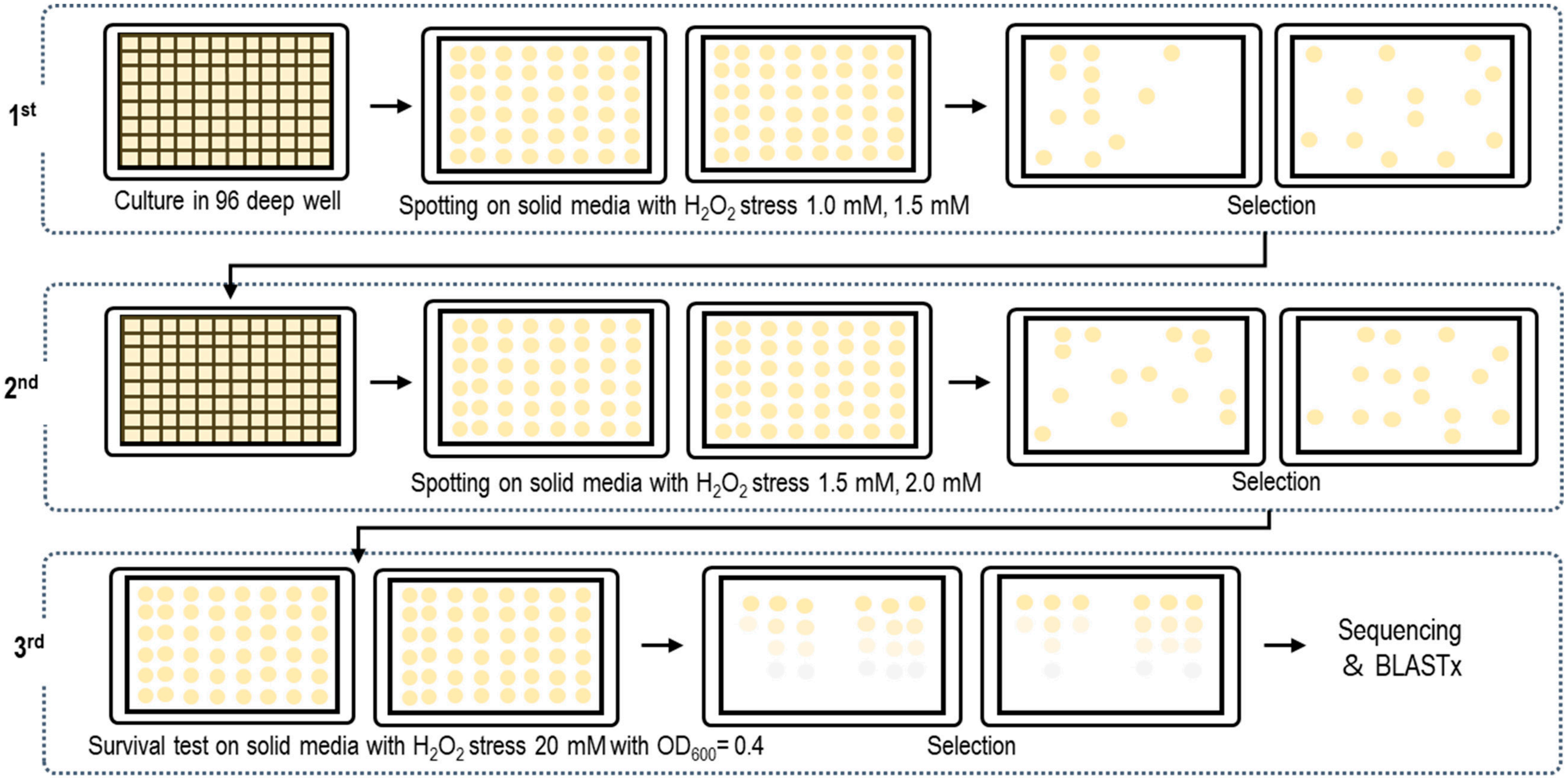

Supple Fig. S1. Screening of positive clones by hydrogen peroxide. *E.coli* (XL1-Blue) containing recombinant DNA fragments were incubated in liquid medium with 50 µg/mL kanamycin and spotting on 1.0, 1.5, and 2.0 mM H_2O_2 in 1st, 2nd screening step, 20 mM H_2O_2 for 1 hr in 3rd screening.

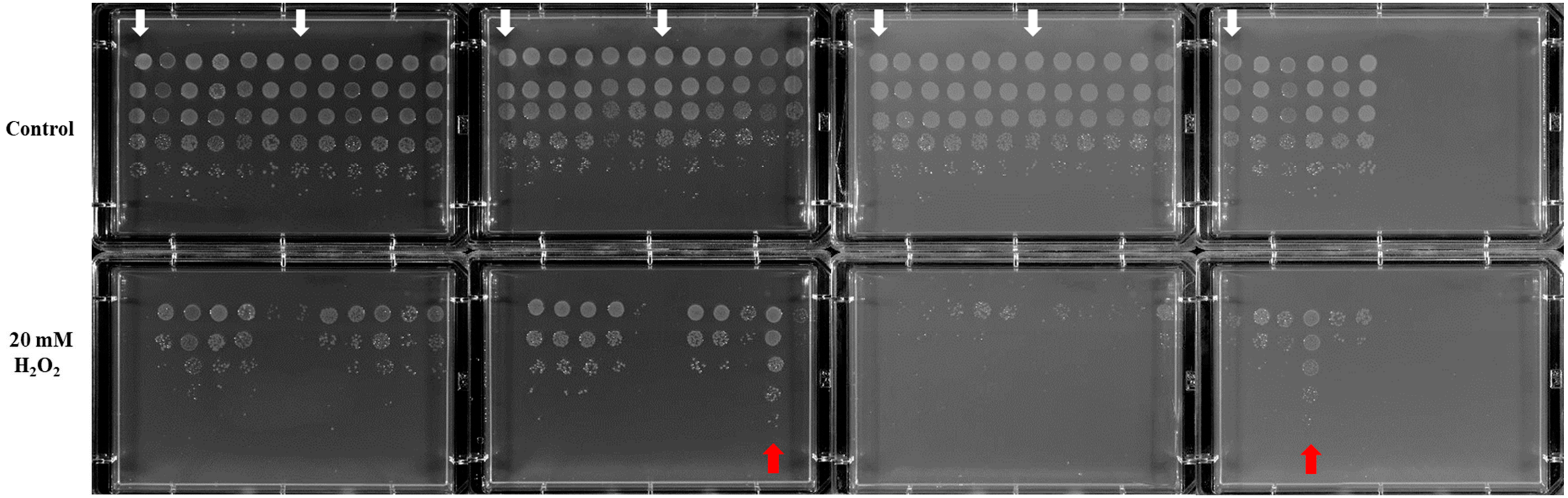

Supple Fig. S2. 3rd screening selection. The white arrows indicate *E.coli* harboring empty vector as control; The red arrows indicate selected E.coli harboring *D. proteolyticus* genes with outstanding oxidative stress resistance. Adjusting cell OD_{600}= 0.4

TCS HK	TCS-RR	DNA binding domain
Deipr_0128	Deipr_0129	OmpR family
Deipr_0376	Deipr_0377	-
Deipr_0799	Deipr_0800	NarL/FixJ family
Deipr_0870	Deipr_0871	NarL/FixJ family
Deipr_1062	Deipr_1061	-
Deipr_1165	Deipr_1167	NarL/FixJ family
Deipr_1504	Deipr_1503	OmpR family
Deipr_1922	Deipr_1923	OmpR family
Deipr_1967	Deipr_1968	OmpR family
Deipr_2675	Deipr_2674	OmpR family
Deipr_2694	Deipr_2693	OmpR family

Orphan HK	Additional domain
Deipr_0252	GAF domain
Deipr_0865	-
Deipr_1801	PAS/PAC domain
Deipr_2217	GAF domain
Deipr_2254	-
Deipr_2523	PAS/PAC domain

Orphan RR	Domain
Deipr_0059	-
Deipr_0147	OmpR family
Deipr_0158	OmpR family
Deipr_0764	wHTH and BTAD domain
Deipr_1307	OmpR family
Deipr_2630	-

HK : Histidine kinase
RR : Response regulator

Core gene (>50%)
Accessory gene (<50%)

Supple Fig. S3. Core and accessory analysis of two component system, orphan histidine kinase and orphan response regulators in D. *proteolyticus.* Core and accessory genes were determined based on the homology (50%) and functional domains were analyzed using interpro database.
